# The Relationship between CT Angiography Collateral Score and Biochemical Parameters during Acute Ischemic Stroke Caused by Middle Cerebral Artery Infarct

**DOI:** 10.3390/jcm13082443

**Published:** 2024-04-22

**Authors:** Halil Güllüoğlu, Hasan Armağan Uysal, Erkan Şahin

**Affiliations:** 1 Department of Neurology, Izmir Ekonomi University Medical Point Hospital, Izmir 35575, Turkey; druysalarmagan@mph.com.tr; 2 Department of Radiology, Izmir Ekonomi University Medical Point Hospital, Izmir 35575, Turkey; erkan.sahin@mph.com.tr

**Keywords:** acute ischemic stroke, MCI, stroke, CT angiography

## Abstract

**Background/Objectives**: Collateral development after AIS is important for prognosis and treatment. In this study, we aimed to investigate the relationship and correlation between biochemical parameters and CT angiography collateral score within the first 9 h and its effect on the neurological outcomes of patients with AIS due to MCA infarction. **Methods**: A total of 98 patients with MCA infarction were hospitalized for diagnosis and treatment after undergoing CT angiography within 9 h of suffering a stroke. Demographic data, admission biochemical parameters, hospitalization data, and discharge NIHSS scores were recorded. Souza’s scoring system for collateral distribution was used to evaluate collaterals. Souza CS system and clinical disability comparison outcomes identified. **Results**: According to the Souza CS system, 13 patients were in the malignant profile category, and 85 patients were in the good profile category. The NIHSS value of patients with a malignant profile was 27, while the mean NIHSS value of patients with a good profile was 9. There was a statistically significant difference in uric acid, total cholesterol, triglyceride, HDL cholesterol, CRP, hsCRP, D-Dimer, troponin I, vitamin B12, fibrinogen, NSE, homocysteine, aPTT, and INR levels according to collateral distribution. **Conclusions**: This study demonstrates that biochemical parameters can influence the distribution of malignant and benign collaterals in AIS independent of age and gender.

## 1. Introduction

Stroke is the third-most-common cause of severe disability and death in all age groups in the worldwide [1]. Ischemic stroke is the most common of all strokes and arterial occlusion (due to thrombus or embolism), and hypoperfusion or hypoxia are the most important problems leading to this condition [2].

Inflammation associated with acute ischemic stroke (AIS) affects infarct size and early neurological deterioration by forming the main mechanism by which cells in the penumbra degenerate [3]. In the AIS process, biochemical parameter levels can be correlated regardless of size and site of the AIS. If this relationship is adequately defined, biochemical parameters may have positive effects in terms of both reducing complications and neurological aspects in relation to the treatment of AIS [4,5,6,7].

Collateral development after an AIS has implications for the prognosis and treatment of these patients. After middle cerebral artery (MCA) occlusion, the leptomeningeal or pial collaterals that connect the distal segments of the MCA to the distal branches of the anterior cerebral artery (ACA) and posterior cerebral artery (PCA) allow ischemic penumbra blood supply. Early and adequate collateral development is associated with smaller infarcts and better neurologic outcomes [8,9]. Although magnetic resonance imaging angiography and catheter angiography modalities are also used to evaluate collaterals, CT angiography (CTA) applications after an AIS are a technique that can be applied safely and quickly in the emergency department [10]. Therefore, several CTA scores have been described so far for the assessment of collateral status in relation to AIS [11].

Based on this, it may be possible to easily use the biochemical markers examined during the admission of patients diagnosed with an MCA infarction and the radiological scores evaluated in the emergency department as a compact marker.

Our aim is to determine the relationship and correlation between biochemical parameters and CTA for collateral score among patients with AIS caused by an MCA infarct in the first 9 h and investigate the effect of this condition on neurological outcomes.

## 2. Materials and Methods

This retrospective, single-center study was conducted between July 2021 and July 2023 in accordance with the Declaration of Helsinki. The research was authorized by the local ethics committee (Izmir Bakırçay University Non-Interventional Clinical Research Ethics Committee, 2023/1116-1096). All patients gave written informed consent to participate in the study and for their data to be used for research.

### 2.1. Patients

A total of 98 patients who had suffered an MCA infarction and been hospitalized for diagnosis and treatment after undergoing CT angiography within 9 h were analyzed. The Dose Escalation of Desmoteplase in Acute Stroke (DEDAS) study further evaluated the safety and effectiveness of IV desmoteplase for patients with perfusion/diffusion mismatch 3 to 9 h after acute stroke onset [12]. Based on this, we included patient evaluations accompanied by imaging data obtained within 9 h (at the latest) in our study. Inclusion criteria were as follows: (1) All enrolled patients had suffered their first stroke; (2) were over the age of 18; (3) had MCA occlusion determined via CTA; (4) had an onset time within 9 h and an MCA infarct diagnosed via CTA within 9 h; (5) had a National Institutes of Health Stroke Scale (NIHSS) score ranging from 5 to 24 on admission; (6) and had been administered the required blood test for all biochemical parameters within 9 h. Exclusion criteria were as follows: (1) Patients who had suffered a transient ischemic attack, cerebral hemorrhage, or hemorrhagic transformation and (2) history of another type of bleeding, infectious disease, demyelinating disease, rheumatic disease, vasculitis, malignancy, and/or chronic renal or liver failure. The patients’ current comorbidities and the drugs they were using or have used (including antiaggregant/anticoagulant drugs) were noted. Information pertaining to demographic data, admission biochemical parameters (i.e., glucose, urea, creatinine, uric acid, total cholesterol, triglyceride, high-density lipoprotein cholesterol (HDL cholesterol), low-density lipoprotein cholesterol (LDL cholesterol), C-reactive protein (CRP), high-sensitivity C-reactive protein (hsCRP), hemoglobin A1c (HbA1c), D-Dimer, erythrocyte sedimentation rate 1 h (ESR 1 h), troponin I, vitamin B12, folic acid, vitamin D3 25-Hydroxyvitamin D (25-OH Vit D3), procalcitonin (PCT), neuron-specific enolase (NSE, where NSE levels were determined using the electrochemiluminescence immunoassay (ECLIA) method), creatine kinase-MB (CK-MB), homocysteine, activated partial thromboplastin time (aPTT), prothrombin time (PT), international normalized ratio (INR), hospitalization, and discharge NIHSS scores (National Institutes of Health Stroke Scale) were recorded. The NIHSS scores were classified as minor (0–5), moderate (5–15), severe (15–20), or very severe (20–42).

### 2.2. CT Protocol

Scanning protocols were performed using a GE Health Care (United States-2021) 512-slice CT scanner. All patients included in the study initially underwent standard non-contrast CT. A total of 50 mL of contrast material (iohexol, Omnipaque, 300 mg of iodine/mL) was used at 5 mL/s for CTA extraction. Multiplane 7 mm maximum intensity projection (MIP) reconstructions and 4 mm axial reformats or CTA source images were acquired. CTA examinations were evaluated independently by an experienced neuroradiologist. Reviewers were blinded in terms of patients’ demographic data, clinic information, imaging follow-up, and treatment.

**Figure 1:**
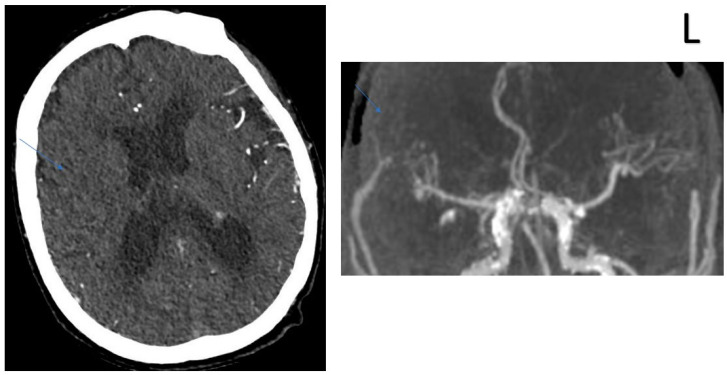
COLLATERAL SCORE: 0–80-year-old female patients. Right MCA infarction. NIHH Score: 23. CT angiography was performed 3.5 h after the acquisition of clinical findings. **Left** image, “Subtracted MIP image obtained from arterial phase images”. **Right** image, “Raw image in the axial plane obtained in the arterial phase after contrast injection”. Arrows represent collateral score area.

**Figure 2:**
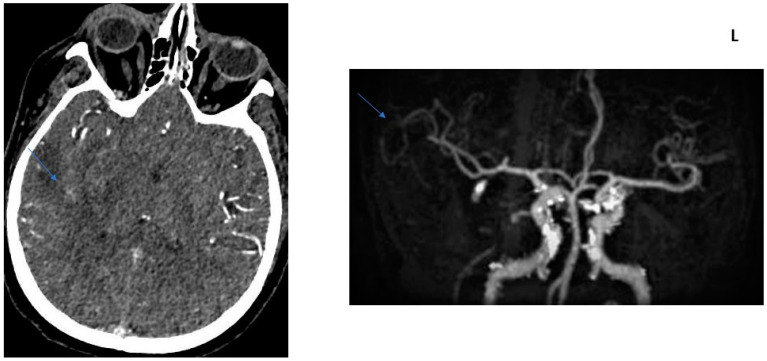
COLLATERAL SCORE: 1–68-year-old female patients. Right MCA infarction. NIHH Score: 14. CT angiography was performed 4.5 h after the acquisition of clinical findings. **Left** image, “Subtracted MIP image obtained from arterial phase images”. **Right** image, “Raw image in the axial plane obtained in the arterial phase after contrast injection”. Arrows represent collateral score area.

**Figure 3:**
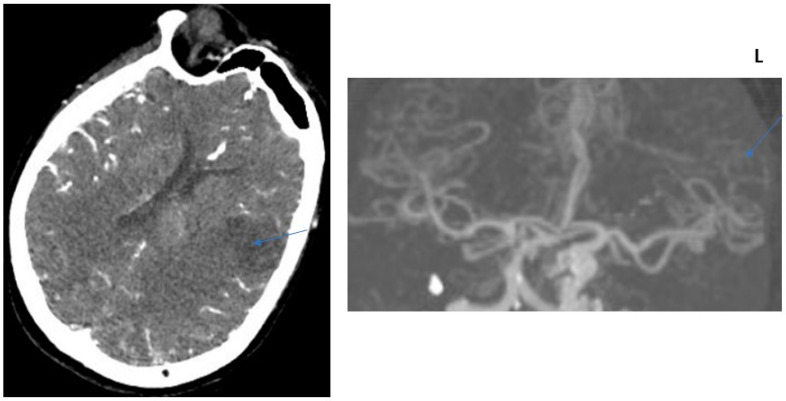
COLLATERAL SCORE: 2–71-year-old female patients. Left MCA infarction. NIHH Score: 18. CT angiography was performed 6 h after the acquisition of clinical findings. **Left** image, “Subtracted MIP image obtained from arterial phase images”. **Right** image, “Raw image in the axial plane obtained in the arterial phase after contrast injection”. Arrows represent collateral score area.

**Figure 4:**
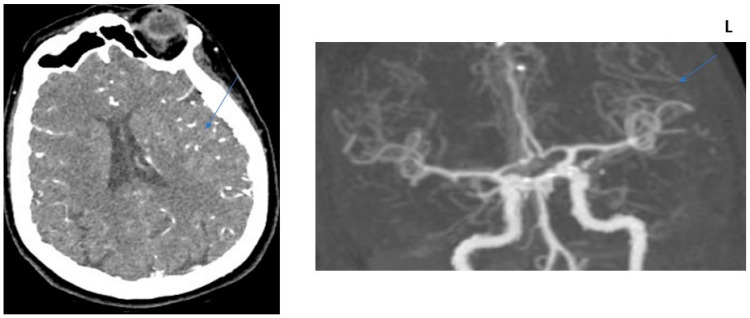
COLLATERAL SCORE: 3–75-year-old male patients. Left MCA infarction. NIHH Score: 9. CT angiography was performed 5 h after the acquisition of clinical findings. **Left** image, “Subtracted MIP image obtained from arterial phase images”. **Right** image, “Raw image in the axial plane obtained in the arterial phase after contrast injection”. Arrows represent collateral score area.

**Figure 5:**
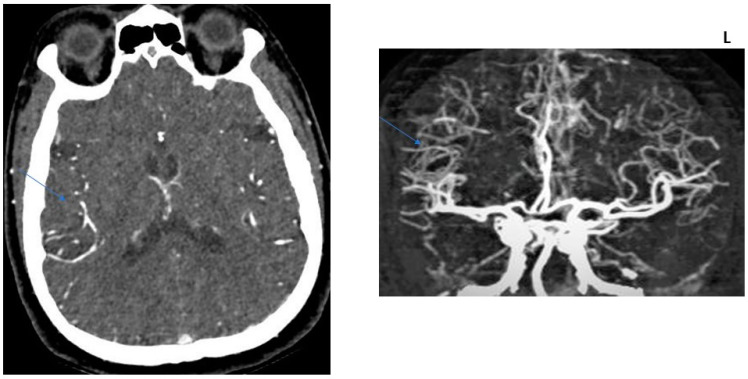
COLLATERAL SCORE: 4–72-year-old female patient. Right MCA infarction. NIHH Score: 12. CT angiography was performed 5.5 h after the acquisition of clinical findings. **Left** image, “Subtracted MIP image obtained from arterial phase images”. **Right** image, “Raw image in the axial plane obtained in the arterial phase after contrast injection”. Arrows represent collateral score area.

For the evaluation of CTA collaterals, Souza et al.’s [13] CTA collateral score (CS) system was used, wherein 0 = absent collaterals > 50% of an M2 territory; 1 = diminished collaterals > 50% M2 territory; 2 = diminished collaterals < 50% M2 territory; 3 = collaterals equal to contralateral side; and 4 = increased collaterals. Patients were dichotomized into 2 categories: CS = 0 (malignant profile) and CS > 0 (good profile) (Figure 1, Figure 2, Figure 3, Figure 4 and Figure 5).

### 2.3. Statistical Analysis

SPSS 25.0 (IBM Corporation, Armonk, NY, USA) and Medcalc 14 (Acacialaan 22, B-8400 Ostend, Belgium) were used to analyze the variables. The conformity of the data to the normal distribution was evaluated with the Shapiro–Wilk and Shapiro–Francia tests, while the homogeneity of variance was evaluated with the Levene test. Mann–Whitney U test with Monte Carlo results was used to compare two independent groups with each other according to quantitative variables. In the comparison of categorical variables, Pearson’s Chi-Square test was tested with Monte Carlo Simulation technique. Since there were too many significant variables, variable selection was performed using the Backward method of the logistic regression test. For the relationship between the selected numerical variables, the classification of the cut-off value calculated according to the variables, and the actual classification, sensitivity, specificity, and positive predictivity and negative prediction predictivity ratios were analyzed and expressed using ROC (Receiver Operating Curve) curve analysis. While quantitative variables are expressed as means (standard deviations) and medians (minimum–maximum) in the tables, categorical variables are shown as n (%). The variables were analyzed at 95% confidence level, and a p value less than 0.05 was considered significant.

## 3. Results

Fifty-six of the patients were males (57.1%), and forty-two were females (43.9%), with an age range of fifty-four to eighty-five (with a mean age of seventy-one). The mean time of admission to the emergency department was 4.57 (1.56) h, the mean CTA time was 5.1 (0.45) h, and the mean blood test time was 5.01 (0.12) h (Table 1). The mean NIHSS score upon arrival was 9.53 (10.5). While the cause of stroke was large artery atherosclerosis (thrombus/embolus) in 28 patients, cardioembolism (high–medium risk) in 27 patients, and small-artery occlusion (lacunar infarction) in 15 patients, the cause could not be determined for 28 patients. The body mass index (BMI) of the patients was 23.9–39.9 (mean: 33.65). While 6 of the patients had normal body weights, 22 patients had pre-obesity, 34 patients were grade 1 obese, and 36 patients were grade 2 obese (Table 1).

The pulse frequency rates of the patients when they came to the emergency room were between a minimum of 65/min and a maximum of 148/min (mean: 111/min). While the mean systolic blood pressure was 160.42 mmHg, the mean diastolic blood pressure was 89.79 mmHg. The minimum and maximum blood glucose levels of the patients when they were admitted to the emergency department were 109 mg/dL and 276 mg/dL (mean 159 mg/dL). A total of 88 (89.8%) patients had hypertension. These patients had been suffering from hypertension for a minimum of 3 years and a maximum of 36 years (mean: 13 years). In total, 51 patients (52%) had diabetes mellitus, suffering from this illness for a minimum of 4 years and a maximum of 21 years (mean: 11 years). Coronary artery disease was present in 60 patients (61.2%), suffering from this illness for a minimum of 3 years and a maximum of 17 years (mean: 8.5 years). A total of 60 patients were hyperlipidemic, and hyperlipidemia was present for a minimum of 5 years and a maximum of 23 years (mean: 10.5 years). A total of 29 of the patients (29.6%) had a history of cardiac arrhythmia. A total of 64 patients had a history of antiaggregant use (65.3%), and 29 patients had a history of anticoagulant use (29.6%). In total, 43 patients (43.9) used alcohol, with a minimum use of 10 years and a maximum use of 45 years (mean: 26 years). A total of 63 patients (64.3%) were smokers. The minimum duration of smoking was 10 years, and the maximum was 45 years (mean: 28 years).

While the body mass index was 29.9 (23.9–37.6) in the malignant profile, it was 34.1 (23.9–39.9) in the good profile (*p* = 0.012). Upon arrival at the emergency room, the patients’ pulse rates were 79/min (65–93/min) in the malignant profile and 115/min (65–148/min) in the good profile (*p* < 0.001). The mean duration of coronary artery disease in patients in the malignant profile group was 14 years (9–17 years), while it was 8 years (3–14/years) in the good profile group. Malignant profile patients were hyperlipidemic for an average of 13 years (10–16 years), whereas patients with a good profile were hyperlipidemic for 9 years (5–23 years) (*p* = 0.012). While none of the patients with a malignant profile had a history of cardiac arrhythmia, all of the patients with a history of cardiac arrhythmia (29 patients) aligned with the good profile (*p* = 0.009). Correspondingly, the use of anticoagulants was only positive for patients with a good profile with a history of cardiac arrhythmia (malignant: none; good: 29 patents) (*p* = 0.009) (Table 1).

According to the Souza CS measurements, 13 (13.3%) individuals had a CS of 0, 13 (13.3%) had a CS of 1, 20 (20.4%) had a CS of 2, 10 (10.2%) had a CS of 3, and 42 (42.8%) had a CS of 4. According to the Souza CS system, 13 patients (13.3%) aligned with the malignant profile, and 85 patients (86.7%) were in the good profile category (Table 1).

The Souza CS system and clinical disability comparison outcomes were identified. Accordingly, the mean NIHSS value of patients with a malignant profile was 27 (with a range from 25 to 30), while the mean NIHSS value of patients with a good profile was 9 (with a range from 3 to 31), and there was a statistically significant difference (*p* < 0.001) (Table 1).

Souza CS system and biochemical parameter analysis comparison outcomes were identified. The mean blood glucose levels at the time of admission to the emergency department were 159 mg/dL (113–196) in patients with a malignant profile and 159 mg/dL (109–276 mg/dL) in patients with a good profile (*p* = 0.526). The urea values were 43.8 mg/dL (26.2–55.6 mg/dL) in the malignant profile and 44.9 mg/dL (21.9–74.6 mg/dL) in the good profile (the normal value is 17.1–49.2 mg/dL) (*p* = 0.748). The creatinine values were 1.07 mg/dL (0.69–1.29 mg/dL) in the malignant profile and 1.13 mg/dL (0.71–1.39 mg/dL) in the good profile (the normal value is 0.72–1.25 mg/dL) (*p* = 0.314). The uric acid values were 9.9 mg/dL (7.9–14.6 mg/dL) in the malignant profile and 8.2 mg/dL (5.9–14.3 mg/dL) in the good profile (the normal value is 3.5–7.2 mg/dL) (*p* < 0.001). The total cholesterol values were 319 mg/dL (251–345 mg/dL) in the malignant profile and 279 mg/dL (213–376 mg/dL) in the good profile (the normal value is 0–200 mg/dL) (*p* = 0.012). The triglyceride values were 236 mg/dL (173–276 mg/dL) in the malignant profile and 179 mg/dL (143–281 mg/dL) in the good profile (the normal value is 0–150 mg/dL) (*p* < 0.001). The HDL cholesterol values were 35 mg/dL (25–46 mg/dL) in the malignant profile and 43 mg/dL (25–81 mg/dL) in the good profile (the normal value is 40–60 mg/dL) (*p* < 0.001). The LDL cholesterol values were 171 mg/dL (148–198 mg/dL) in the malignant profile and 177 mg/dL (123–231 mg/dL) in the good profile (the normal value is 0–130 mg/dL) (*p* = 0.944). The CRP values were 9 mg/L (7–12 mg/L) in the malignant profile and 6 mg/L (3–10 mg/L) in the good profile (the normal value is 0–5 mg/L) (*p* < 0.001). The hsCRP values were 9.13 mg/L (3.12–11.93 mg/L) in the malignant profile and 4.85 mg/L (2.35–11.89 mg/L) in the good profile (the normal value is 0.10–2.80 mg/L) (*p* = 0.014). The HbA1c values were 6.98% (5.45–10.48%) in the malignant profile and 6.37% (5.06–12.8%) in the good profile (*p* = 0.857). The D-Dimer values were 1020 µg/L (559–1286 µg/L) in the malignant profile and 736 µg/L (451–1369 µg/L) in the good profile (the normal value is 0–550 µg/L) (*p* = 0.001). The ESR 1 h values were 25 mm (11–39 mm) in the malignant profile and 19 mm (10–37 mm) in the good profile (the normal value is 0–20 mm) (*p* = 0.081) (Table 2).

The troponin I values were 81.4 pg/mL (17.5–110.5 pg/mL) in the malignant profile and 56.4 pg/mL (19.9–99.6 pg/mL) in the good profile (the normal value is 0–34.2 pg/mL) (*p* = 0.034). The vitamin B12 values were 192 pg/mL (165–299 pg/mL) in the malignant profile and 251 pg/mL (103–432 pg/mL) in the good profile (the normal value is 187–883 pg/mL) (*p* = 0.012). The folic acid values were 2.6 ng/mL (1.1–8.6 ng/mL) in the malignant profile and 3.7 ng/mL (1.7–9.9 ng/mL) in the good profile (the normal value is 3.1–20.5 ng/mL) (*p* = 0.073). The 25-OH Vit D3 values were 28 ng/mL (13–49 ng/mL) in the malignant profile and 31 ng/mL (10–62 ng/mL) in the good profile (the normal value is 40–100 ng/mL) (*p* = 0.631). The PCT values were 0.9 ng/mL (0.7–1.8 ng/mL) in the malignant profile and 0.8 ng/mL (0.1–2.9 ng/mL) in the good profile (the normal value is 0–0.5ng/mL) (*p* = 0.164). The fibrinogen values were 9.55 g/L (6.85–10.98 g/L) in the malignant profile and 6.93 g/L (3.86–9.95 g/L) in the good profile (the normal value is 2.1–4 g/L) (*p* < 0.001) (Table 2).

The NSE values were 19.9 ng/mL (15.9–25.6) in the malignant profile and 15.9 ng/mL (9.3–21.8 ng/mL) in the good profile (the normal value is 0–16.9 ng/mL) (*p* < 0.001). The CK-MB values were 18.8 U/L (11.9–27.1 U/L) in the malignant profile and 20.9 U/L (12.4–29.9 U/L) in the good profile (the normal value is 25.0 U/L) (*p* = 0.126). The homocysteine values were 21.9 umol/L (19.3–23.6 umol/L) in the malignant profile and 18.8 umol/L (10.9–31.9 umol/L) in the good profile (the normal value is 5.46–16.2 umol/L) (*p* = 0.001). The aPTT values were 25.6 s (20.3–27.8 s) in the malignant profile and 26.8 s (20.1–33.9 s) in the good profile (the normal value is 22.7–31.8 s) (*p* = 0.045). The PT values were 13.2 s (10.8–14.4 s) in the malignant profile and 12.6 s (10.1–15.9 s) in the good profile (the normal value is 10.4–13 s) (*p* = 0.455). The INR values were 1.1 C (0.7–1.5 C) in the malignant profile and 1.3 s (0.6–3.1 C) in the good profile (the normal value is 0.8–1.2 C) (*p* = 0.062). The Souza CS System did indicate that there were statistically significant differences in uric acid, total cholesterol, triglyceride, HDL cholesterole, CRP, hsCRP, D-Dimer, troponin I, vitamin B12, fibrinogen, NSE, homocysteine, aPTT, and INR levels (Table 2).

## 4. Discussion

Collateral circulation, assessed on a venous and arterial (primary and secondary) basis, is defined as additional vascular pathways that provide the continuation of blood flow to the target tissue during and after AIS. The primary arterial collateral system refers to the short artery segments in the Circle of Willis, while the secondary arterial collateral system refers to the pial (leptomeningeal) collaterals. Primary arterial collaterals connect the internal carotid system to the vertebrobasilar system or cerebral hemisphere, while secondary arterial collaterals (pial collaterals) anastomose between the distal branches of the ACA, MCA, PCA, and the pial surface of the cortex. With pial collaterals, blood flow is provided to an occluded arterial area during and after an AIS from the normal-circulation arterial system [14,15,16,17].

CTA is used to determine collateral flow by many people, clinics, and centers because it is non-invasive, easy to use, and easily accessible and allows rapid evaluation, among other things [10,11,18]. Although many scoring systems (the Alberta Stroke Program Early CT Score (ASPECTS), Christoforidis collateral score, Miteff collateral score, Maas collateral score, Tan collateral score, Careggi collateral score, and Souza collateral score) have been used to evaluate the CTA collateral system, there is still no consensus on scoring [9,13,18,19,20,21,22]. Patients with a malignant profile are less likely to benefit from revascularization treatment due to the risk of having a large infarct volume before treatment. Based on this prediction, such patients can be identified, and treatment decisions can be influenced. Puetz et al. [23] used CTA-based imaging to define the malignant stroke population, employing a 20-point score combining the ASPECT score and clot burden. The resulting population comprised 114 anterior circulation stroke patients treated with IV tPA within three hours. The authors found 24 patients (21%) with a combined score of 10 (malignant profile) and reported that half of the patients died.

We used the Souza CS system in our study. Because this scoring system includes a simple, reliable, and easily applicable rating, it may also provide faster real-time assessments and be more suitable for patient selection for intra-arterial therapy. In addition, a highly specific malignant collateral profile was identified for patients with large initial infarcts who were at high risk of having a poor long-term outcome. Patients with absent collaterals in >50% of 1 MCA M2 division territory (CS system 0) have a poor clinical outcome. Collateral score may predict final infarct size as well as functional outcome, and for patients with a malignant profile, infarct size is large, and functional outcome is poor. Bang et al. [24] reported that patients with a good angiographic profile had higher recanalization rates and less infarct growth after intra-arterial treatment. Angermaier et al. [25] highlighted that CTA collateral grade was an independent predictor of final infarct volume among stroke patients treated with endovascular therapy. Rosenthal et al. [26] evaluated the effect of CTA collaterals on patients with and without complete recanalization and reported that the positive effects were exhibited by patients who were not fully recanalized. Lima et al. suggested that there may be a greater beneficial effect of collaterals for untreated patients [18]. In addition to all this literature-derived information, it is clear that more studies on untreated patients are needed, as malignant and good profiles of the collateral circulation should have a more important role in determining infarct volume and clinical outcome.

In our study, we found statistical differences in terms of NIHSS score, BMI, pulse frequency rate, duration of coronary artery disease, duration of hyperlipidemia, history of cardiac arrhythmia, and history of anticoagulant use among clinical evaluations. In terms of biochemical parameters and biochemical parameters, there were statistical differences in uric acid, total cholesterol, triglyceride, HDL cholesterol, CRP, hsCRP, D-dimer, troponin, vitamin B12, fibrinogen, NSE, homocysteine, aPTT, and INR levels. To the best of our knowledge, our study is the first study in the literature to evaluate the above clinical and biochemical parameters together. In this study, we demonstrated the effects of the above-mentioned biochemical parameter levels on the distribution of malignant and good profile collaterals in MCA infarction independent of age and gender and its effects on clinical outcome and prognosis.

Previous studies in the literature have shown that uric acid can have both positive and negative effects on the outcomes of AIS patients [27,28,29,30,31]. Liu et al. [28] reported that extremely low or high uric acid levels have a negative effect on prognosis. In their study, they emphasized that low levels of uric acid (<4.1 mg/dL) may lead to adverse clinical outcome and death among men, while high levels of uric acid (>6.6 mg/dL) may predict death among women. For our patients, we observed that increasing uric acid values increased the displacement of the collateral distribution towards the malignant profile and worsened prognosis.

Previous studies in the literature have highlighted that elevated plasma hsCRP levels are independently associated with adverse clinical outcomes after acute ischemic stroke [32,33]. In addition, Gu et al. [33] recently reported that stroke recurrence was associated with less than 20% of the association between hsCRP and functional outcomes at 90 days among patients with ischemic stroke, suggesting that more emphasis should be placed on novel anti-inflammatory therapy to improve functional outcomes. In our study, we found that both CRP and hsCRP levels were higher in patients with a malignant profile of the CS distribution.

In the literature, there are not enough studies on the mechanisms of how vitamin B12 deficiency affects functional outcomes in relation to AIS. In addition, it has been observed that low vitamin B12 levels are a risk factor for ischemic stroke, a finding that is persistently reported in the literature [34]. In our study, vitamin B12 levels were statistically significantly lower in the malignant profile CS.

Although different correlations between infract volume and NIHSS scores have been reported in the literature, it has been emphasized that NSE may be a diagnostic and prognostic biomarker for AIS [7,35,36]. In our patients, NSE was statistically significantly elevated in patients with malign CS.

Elevated homocysteine levels are a well-known risk factor for having an AIS [37,38,39]. Our results showed increases in homocysteine levels that are in line with the literature. In addition, there were statistically significantly higher levels in the malignant profile compared to the good profile.

Our study has some inevitable limitations. Considering that the patients presented to a single center and that their anamnesis included the first 9 h window period, the treatments received by the patients during first aid administration before admission were not taken into account. It was anticipated that this would not have a direct impact on the study results. In addition, it was not possible to evaluate in detail the severity of dysregulated diabetes mellitus, uncontrolled hypertension, and accompanying cardiovascular diseases affecting the clinical statuses of the patients. However, the lack of difference in statistical analysis is an important finding. As a result of our study, it is thought that patients will provide accurate information for prognoses and new potential treatment options with which to examine the correlations between CT angiography collateral scores, biochemical parameters, neurological examination, and NIHSS scores.

## 5. Conclusions

Our results showed the association of some biochemical parameters with collateral distribution, suggesting that this distribution may be a prognostic indicator for the development of clinical complications in cerebral ischemia. Assessment of different plasma levels of biochemical parameters in AIS patients may be predictive of both the nature of the collateral distribution of ischemia and clinical outcome and prognosis.

## Figures and Tables

**Table 1:** Comparison of clinical data and Souza CTA collateral score system.

	CTA	CS	
	<1	≥1	
	(*n* = 13)	(*n* = 85)	*p*
Age, median (min/max)	75 (60/82)	71 (54/85)	0.120 ^u^
Gender (male), *n* (%)	9 (69.2)	47 (55.3)	0.386 ^c^
Mean time of admission to the emergency department, median (min/max)	3.5 (2.5/6.5)	4.5 (2/9)	0.066 ^u^
Causes of stroke, *n* (%)			
Large-artery atherosclerosis (embolus/thrombosis)	13 (100)	15 (17.6)	
Cardioembolism (high-risk/medium-risk)	0 (0)	27 (31.8)	
Small-vessel occlusion (lacune)	0 (0)	15 (17.6)	
Stroke of other determined etiology	0 (0)	0 (0)	
Stroke of undetermined cause	0 (0)	28 (32.9)	
BMI, median (min/max)	29.9 (23.9/37.6)	34.1 (23.9/39.9)	0.012 ^u^
BMI, *n* (%)			0.008 ^ff^
Normal	2 (15.4)	4 (4.7)	
Pre-obesity	5 (38.5)	17 (20)	0.014
Grade 1 obese	4 (30.8)	30 (35.3)	
Grade 2 obese	2 (15.4)	34 (40)	0.011
Grade 3 obese	0 (0)	0 (0)	
NIHSS score, median (min/max)	27 (25/30)	9 (3/31)	<0.001 ^u^
Pulse frequency rates of the patients upon admission to the emergency room, minutes, median (min/max)	79 (65/93)	115 (65/148)	<0.001 ^u^
Mean systolic blood pressure, mmHg, median (min/max)	165 (123/177)	159 (125/204)	0.927 ^u^
Mean diastolic blood pressure, mmHg, median (min/max)	91 (79/105)	89 (69/121)	0.353 ^u^
Blood glucose of the patients upon admission to the emergency room, mg/dL, median (min/max)	159 (113/196)	159 (109/276)	0.526 ^u^
Hypertension, *n* (%)	13 (100)	75 (88.2)	0.350 ^f^
Duration of hypertension, years, median (min/max)	23 (5/36)	12 (3/35)	0.061 ^u^
Diabetes mellitus, *n* (%)	8 (61.5)	43 (50.6)	0.558 ^c^
Duration of diabetes mellitus, years, median (min/max)	13 (7/19)	11 (4/21)	0.440 ^u^
Coronary artery disease, *n* (%)	9 (69.2)	51 (60)	0.563 ^c^
Duration of coronary artery disease, years, median (min/max)	14 (9/17)	8 (3/14)	<0.001 ^u^
Hyperlipidemia, *n* (%)	9 (69.2)	51 (60)	0.563 ^c^
Duration of hyperlipidemia, years, median (min/max)	13 (10/16)	9 (5/23)	0.012 ^u^
History of cardiac arrhythmia, *n* (%)	0 (0)	29 (34.1)	0.009 ^f^
History of antiaggregant use, *n* (%)	9 (69.2)	55 (64.7)	0.999 ^f^
History of anticoagulant use, *n* (%)	0 (0)	29 (34.1)	0.009 ^f^
History of alcohol use, *n* (%)	28 (18/41)	25.5 (10/45)	0.620 ^u^
Duration of alcohol use, years, median (min/max)	28 (18/41)	25.5 (10/45)	0.620 ^u^
History of smoking, *n* (%)	10 (76.9)	53 (62.4)	0.368 ^f^
Duration of smoking, years, median (min/max)	31 (20/45)	28 (10/45)	0.315 ^u^

^u^ Mann–Whitney *U* Test (Monte Carlo), ^f^ Fisher Exact Test (Monte Carlo), ^ff^ Fisher Freeman Halton Test (Monte Carlo), and ^c^ Pearson Chi-Square Test (Monte Carlo).

**Table 2:** Comparison of biochemical parameters and serum biomarkers and the Souza CTA collateral score system.

	CTA	CS	
	<1	≥1	
	(*n* = 13)	(*n* = 85)	*p*
Urea (normal, 17.1–49.2 mg/dL), median (min/max)	43.8 (26.2/55.6)	44.9 (21.9/74.6)	0.748 ^u^
Creatinine (normal, 0.72–1.25 mg/dL), median (min/max)	1.07 (0.69/1.29)	1.13 (0.71/1.39)	0.314 ^u^
Uric acid (normal, 3.5–7.2 mg/dL), median (min/max)	9.9 (7.9/14.6)	8.2 (5.9/14.3)	<0.001 ^u^
Total cholesterol (normal, 0–200 mg/dL), median (min/max)	319 (251/345)	279 (213/376)	0.012 ^u^
Triglyceride (normal, 0–150 mg/dL), median (min/max)	236 (173/276)	179 (143/281)	<0.001 ^u^
HDL cholesterol (normal, 40–60 mg/dL), median (min/max)	35 (25/46)	43 (25/81)	<0.001 ^u^
LDL cholesterol (normal, 0–130 mg/dL), median (min/max)	171 (148/198)	177 (123/231)	0.944 ^u^
CRP (normal, 0–5 mg/L), median (min/max)	9 (7/12)	6 (3/10)	<0.001 ^u^
hsCRP (normal, 0.10–2.80 mg/dL), median (min/max)	9.13 (3.12/11.93)	4.85 (2.35/11.89)	0.014 ^u^
HbA1c (%, median (min/max)	6.98 (5.45/10.48)	6.37 (5.06/12.8)	0.857 ^u^
D-Dimer (normal, 0–550 µg/L), median (min/max)	1020 (559/1286)	736 (451/1369)	0.001 ^u^
ESR 1 h (normal, 0–20 mm), median (min/max)	25 (11/39)	19 (10/37)	0.081 ^u^
Troponin I (normal, 0–34.2 pg/mL), median (min/max)	81.4 (17.5/110.5)	56.4 (19.9/99.6)	0.034 ^u^
Vitamin B12 (normal, 187–883 pg/mL), median (min/max)	192 (165/299)	251 (103/432)	0.012 ^u^
Folic acid (normal, 3.1–20.5 ng/mL), median (min/max)	2.6 (1.1/8.6)	3.7 (1.7/9.9)	0.073 ^u^
25-OH Vit D3 (normal, 40–100 ng/mL), median (min/max)	28 (13/49)	31 (10/62)	0.631 ^u^
Procalcitonine (normal, 0–0.5 ng/mL), median (min/max)	0.9 (0.7/1.8)	0.8 (0.1/2.9)	0.164 ^u^
Fibrinogen (normal, 2.1–4 g/L), median (min/max)	9.55 (6.85/10.98)	6.93 (3.86/9.95)	<0.001 ^u^
NSE (normal, 0–16.3 ng/mL), median (min/max)	19.9 (15.9/25.6)	15.9 (9.3/21.8)	<0.001 ^u^
CK-MB (normal, 25.0 U/L), median (min/max)	18.8 (11.9/27.1)	20.9 (12.4/29.9)	0.126 ^u^
Homocysteine (normal, 5.46–16.2 umol/L), median (min/max)	21.9 (19.3/23.6)	18.8 (10.9/31.9)	0.001 ^u^
aPTT (normal, 22.7–31.8 sn), median (min/max)	25.6 (20.3/27.8)	26.8 (20.1/33.9)	0.045 ^u^
PT (normal, 10.4–13 sn), median (min/max)	13.2 (10.8/14.4)	12.6 (10.1/15.9)	0.455 ^u^
INR (normal, 0.8–1.2 C), median (min/max)	1.1 (0.7/1.5)	1.3 (0.6/3.1)	0.062 ^u^

^u^ Mann–Whitney *U* Test (Monte Carlo).

## Data Availability

The datasets analyzed in this study are available from the corresponding author upon reasonable request.
